# Network-Based Analysis of Affected Biological Processes in Type 2 Diabetes Models

**DOI:** 10.1371/journal.pgen.0030096

**Published:** 2007-06-15

**Authors:** Manway Liu, Arthur Liberzon, Sek Won Kong, Weil R Lai, Peter J Park, Isaac S Kohane, Simon Kasif

**Affiliations:** 1 Department of Biomedical Engineering, Boston University, Boston, Massachusetts, United States of America; 2 Department of Cardiology, Children's Hospital, Boston, Massachusetts, United States of America; 3 Children's Hospital Informatics Program at the Harvard-MIT Division of Health Sciences and Technology, Boston, Massachusetts, United States of America; 4 Harvard-Partners Center for Genetics and Genomics, Boston, Massachusetts, United States of America; 5 Center of Biomedical Informatics, Harvard Medical School, Boston, Massachusetts, United States of America; 6 Center for Advanced Genomic Technology, Boston University, Boston, Massachusetts, United States of America; University of Washington, United States of America

## Abstract

Type 2 diabetes mellitus is a complex disorder associated with multiple genetic, epigenetic, developmental, and environmental factors. Animal models of type 2 diabetes differ based on diet, drug treatment, and gene knockouts, and yet all display the clinical hallmarks of hyperglycemia and insulin resistance in peripheral tissue. The recent advances in gene-expression microarray technologies present an unprecedented opportunity to study type 2 diabetes mellitus at a genome-wide scale and across different models. To date, a key challenge has been to identify the biological processes or signaling pathways that play significant roles in the disorder. Here, using a network-based analysis methodology, we identified two sets of genes, associated with insulin signaling and a network of nuclear receptors, which are recurrent in a statistically significant number of diabetes and insulin resistance models and transcriptionally altered across diverse tissue types. We additionally identified a network of protein–protein interactions between members from the two gene sets that may facilitate signaling between them. Taken together, the results illustrate the benefits of integrating high-throughput microarray studies, together with protein–protein interaction networks, in elucidating the underlying biological processes associated with a complex disorder.

## Author Summary

Type 2 diabetes mellitus currently affects millions of people. It is clinically characterized by insulin resistance in addition to an impaired glucose response and associated with numerous complications including heart disease, stroke, neuropathy, and kidney failure, among others. Accurate identification of the underlying molecular mechanisms of the disease or its complications is an important research problem that could lead to novel diagnostics and therapy. The main challenge stems from the fact that insulin resistance is a complex disorder and affects a multitude of biological processes, metabolic networks, and signaling pathways. In this report, the authors develop a network-based methodology that appears to be more sensitive than previous approaches in detecting deregulated molecular processes in a disease state. The methodology revealed that both insulin signaling and nuclear receptor networks are consistently and differentially expressed in many models of insulin resistance. The positive results suggest such network-based diagnostic technologies hold promise as potentially useful clinical and research tools in the future.

## Introduction

Type 2 diabetes mellitus (DM2) is a metabolic disorder characterized by abnormal hepatic glucose output, insulin resistance, and impaired insulin production [1,2]. DM2 has reached epidemic proportions and currently affects about 170 million people worldwide, with the figure projected to more than double by 2030 [3]. The driving force behind the high prevalence of diabetes is the rise of obesity in the population [4]. In the United States alone, a startling 32% of the population is classified as obese [5]. The long-term complications of DM2 include atherosclerotic vascular disease, heart disease, retinopathy, kidney failure, and amputation [3].

DM2 is currently believed to be a multifactorial, complex disease [6–8]. While patients may all exhibit the aforementioned classical set of symptoms, individual cases can vary significantly in their internal cause–effect physiological mechanisms. The same diversity in the biology underlying the disorder also appears among the different animal models. Although they may all exhibit hyperglycemia and insulin resistance to certain degrees, the organisms differ with respects to diet, drug treatment, and gene knockouts.

The framework described in this paper is aimed to address two key questions: (1) Can biological processes be identified that are consistently deregulated in different models of insulin resistance and diabetes and that may be manifested in a tissue-dependent or independent manner? (2) On a higher level, can tissue or condition-specific interaction networks be identified that more precisely characterize different insulin-resistance models and suggest causal mechanisms?

By identifying key biological processes and genes involved in the pathogenesis of diabetes, novel drug targets for the disease and related metabolic disorders such as obesity and metabolic syndrome may be determined.

We began the investigation by focusing on insulin-signaling genes, a natural and well-established candidate for finding a signature set of genes associated with insulin resistance or diabetes [9]. In particular, by examining microarray data, we attempted to detect a statistically significant, transcriptional alteration in a set of insulin-signaling genes in diabetic tissue compared to normal. Surprisingly, using existing analytical methods, we were unable to detect such alterations in microarray data produced in several human studies. Using sophisticated and remarkably sensitive techniques, previous studies identified the oxidative phosphorylation pathway as transcriptionally down-regulated in diabetic muscle tissue compared to normal [10,11]. However, insulin-signaling gene sets were not detected to be transcriptionally altered, using state of the art analyses, more than expected by chance.

Since insulin signaling is a key biological process involved in insulin resistance, our inability to detect its transcriptional alteration in diabetes posed a scientific and methodological challenge. We consequently analyzed a set of microarray datasets, generated by the Diabetes Genome Anatomy Project (DGAP) [12], from different experiments of insulin resistance and diabetes in mouse models and human patients. A number of these experiments directly perturbed critical components of insulin signaling and hundreds of genes were observed differentially expressed in the disease state. Using a well-established and widely used methodology for detecting significantly altered biological processes, we were again unable to detect a significant change in insulin signaling in these experiments.

The inability to detect insulin-signaling changes in both studies can be explained by a number of technical and biological hypotheses. First, perhaps the number of insulin-signaling genes that were transcriptionally deregulated was too few to be considered significant by statistical procedures. Second, perhaps the assembled insulin-signaling gene set used in our analysis did not accurately capture the transcriptional alterations in insulin signaling. Alternatively, it is plausible that the changes in a diabetic state were produced by phosphorylation-mediated signaling that was not detected by transcriptional profiling.

We decided to pursue the first hypothesis and adapted a systems biology perspective. Rather than looking for significant aberrations in expression of individual insulin-signaling genes, we looked for significant aberrations in the collective expression of a set of insulin-signaling genes whose protein products form a connected protein–protein interaction network. This was accomplished using a simple methodology referred to as gene network enrichment analysis (GNEA).

Application of GNEA to the microarray datasets convincingly identified a set of insulin-signaling genes (denoted as IS-HD) as significantly transcriptionally altered in insulin resistance and the DM2 phenotype. Additionally, GNEA identified another transcriptionally altered set of genes containing many nuclear receptor and nuclear receptor cofactors (denoted as NR-HD). The NR-HD set contained such genes as *peroxisome proliferative activated receptor, gamma, coactivator 1 alpha (PPARGC1A,* also known as *PGC1A), peroxisome proliferative activated receptor, gamma (PPARG),* and *hepatocyte nuclear factor 4 alpha 1 (HNF4A),* whose associations with the DM2 phenotype were well established [10,13–17]. To our knowledge, this was the first genome-wide study that addressed the broad transcriptional role of a set of nuclear receptors in different insulin resistance and DM2 models, on the basis of integration of gene-expression data from diverse models of the disorder. In particular, the results demonstrated the consistent deregulation of a set of insulin-signaling genes and a set of nuclear receptors in multiple insulin-resistance models.

## Methods

### GNEA Overview

GNEA aims to identify biological processes that are consistently deregulated across a broad set of microarray experiments associated with different disease models in both human and animal tissues. This differs from conventional statistical methods that typically focus on finding biological processes affected in a single microarray experiment. Similar to gene-set enrichment analysis (GSEA) [18], the approach allows for the inclusion of moderately expressed genes, which may be missed by traditional tests for differential expression.

GNEA is motivated by the following model. The cell is associated with a protein–protein interaction network, and each protein is labeled as belonging to one or more gene sets associated with biological processes or molecular function. This labeling defines overlapping functional subnetworks of related proteins. Such a model can be formalized as a biological context network, and previous studies have shown that the distribution of labels per protein follows a power law distribution [19].

When the cell enters a perturbed state, some subset of the interaction network becomes differentially affected. The hypothesis is that certain functional subnetworks may show significantly altered activity in the perturbed state compared to normal. Such functional subnetworks may be identified using statistical methods such as GNEA.

**Figure 1:**
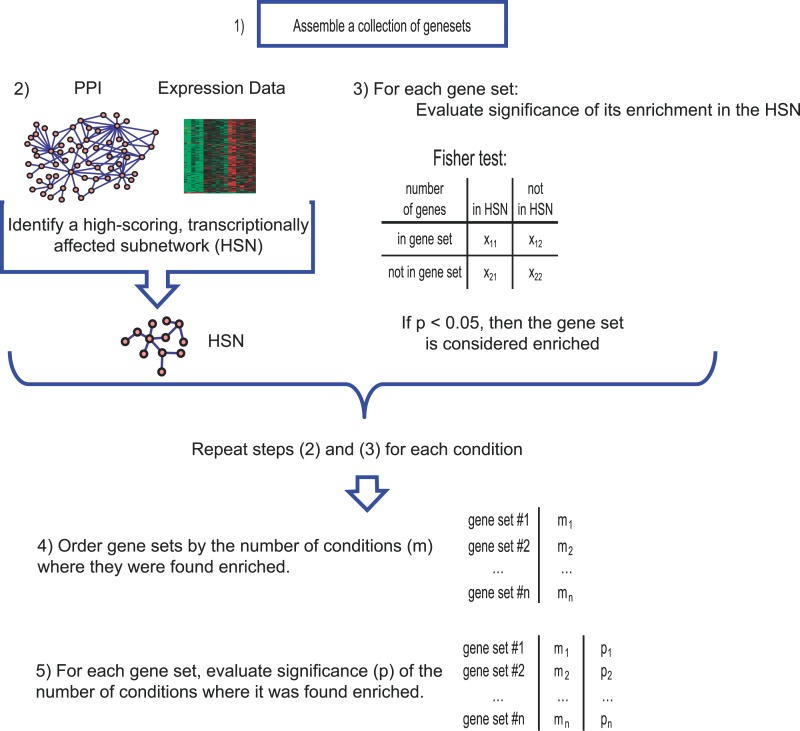
Overview of GNEA (1) A collection of gene sets associated with biological processes was assembled. (2) The relative mRNA expression of every gene in each insulin resistance or diabetes condition was mapped to the associated protein in a global network of protein–protein interactions and a significantly transcriptionally affected subnetwork, HSN, was identified. (3) Each gene set was tested for overrepresentation in each HSN based on a Fisher's exact test. A gene set was considered enriched in the condition if the Fisher's *p*-value fell below 0.05. (4) Gene sets were ordered based on the number of conditions in which they were enriched. (5) For each gene set, the number of conditions in which it was enriched was assigned a *p*-value based on comparison against a background distribution.

The basic framework for GNEA consists of five steps: (1) Assemble a collection of gene sets associated with biological processes or signaling pathways of interest (for example, insulin signaling). (2) Assume an underlying model of cellular processes using a global protein–protein interaction network, imported from the literature. We associate each protein in the interaction network with the relative change in mRNA expression between an insulin resistant or diabetic state and normal condition. Based on the interaction network and gene expressions, we find a subnetwork (designated as the high-scoring subnetwork [HSN]) that is highly transcriptionally affected in the disease state. (3) Evaluate the hypothesis that genes in a given gene set are observed in a higher proportion (i.e., enriched) than expected by chance in the HSN and repeat for each gene set in the assembly. Repeat (2) and (3) for every insulin resistant or diabetic condition compared to normal in the dataset. (4) Order the gene sets of interest based on the number of different HSNs where they appear enriched. (5) For each gene set, assign a *p*-value to the number of conditions where it is enriched. The gene sets with a significant *p*-value are taken as transcriptionally affected across a broad set of diabetes-related models. Consistent with the stated goal of GNEA, gene sets enriched in a few conditions, while potentially interesting in their own right, will not generally be assigned a significant *p*-value (Figure 1).

To assess the effectiveness of GNEA, we also compared its performance against the conventional hypergeometric enrichment test for differentially expressed genes (DEA) and GSEA [18]. The basic outline for DEA is similar to GNEA without the incorporation of protein–protein interaction information. Complete details are given in Figure S1.

### Assembling Gene Sets for Testing with GNEA

We collected and tested two groups of gene sets separately. One group contained gene sets potentially associated with DM2, based on composition of its members, and included IS-HD, NR-HD, two manually curated retinol signaling and metabolism sets, and three additional nuclear receptor gene sets compiled from the nuclear receptor superfamily, labeled nuclear receptors from the HUGO gene nomenclature committee, and the union of the two. The selection of retinol signaling and nuclear receptor gene sets was motivated by previous studies, suggesting a possible connection between retinol signaling, DM2, and related metabolic disorders, as well as by the established roles between individual nuclear receptors and metabolic processes. The IS-HD gene set was taken directly from the Human Protein Reference Database (HPRD) [20], a public compilation of protein–protein interactions. The NR-HD gene set was manually compiled and curated. The other group contained 346 gene sets from gene ontology (GO) categories present in the DGAP expression data that fell within a minimum and maximum size threshold. See Text S2 for details.

### Mapping Gene Expression to a Protein–Protein Interaction Network

The gene-expression data were a compilation of diverse gene-expression datasets of diabetes and insulin resistance models derived from a collection of 361 oligonucleotide microarrays generated by 16 projects for DGAP (see Text S1 for details). Detailed descriptions of each particular experiment along with the expression data can be found on the DGAP website [12]. These experiments had different designs with respect to organism (human or mouse), tissue type (adipose, liver, muscle, and pancreas), and models of insulin resistance and diabetes.

The data were grouped into 67 different gene-expression profiles, collectively designated as DGAP conditions, following the designs and setups of the original experiments. A total of 65 of these 67 conditions directly compared between an insulin resistant or diabetic phenotype to a control phenotype within an experiment. The remaining two conditions compared between insulin-dependent response phenotypes under fasting and fed states within an experiment (see Datasets S1, S2, and S3 for details). The expression profiles of all the genes in the 67 conditions were designated as the DGAP expression data.

The global protein–protein interaction network was obtained from the HPRD. For each of the DGAP conditions, the *p*-value (significance of expression change between insulin resistant or diabetic and control state) of every gene was mapped onto the corresponding protein in the network. This generated a set of condition-specific, protein–protein interaction networks wherein each protein is labeled with a *p*-value for each condition (see Text S3 for details). In other words, while the network interactions were static, the *p*-values associated with the genes and proteins changed per condition. It is important to note that genes with no corresponding proteins in the protein–protein interaction network were not included in the analysis. Similarly, proteins in the HPRD without a corresponding gene in the DGAP expression data were excluded from the protein–protein interaction network along with all associated interactions.

For each gene set, annotation of the corresponding proteins in the condition-specific, protein–protein interaction network produced a functional subnetwork.

### High-Scoring Networks: Putative Noisy Molecular Models of Disease

A network search algorithm was used to identify an HSN per condition-specific protein–protein interaction network. The algorithm attempted to identify an interaction subnetwork whose cumulative expression level (based on an average of the expression for the individual nodes) was differentiated from the background. It is important to note that the subnetwork allowed the inclusion of genes that were not found as individually significant but that were connected to other significant genes through protein–protein interactions. This feature allowed genes with known interactions to other transcriptionally active genes to be included, even if they did not exhibit high transcriptional activity themselves. Therefore, such a subnetwork containing a large number of transcriptionally affected members and associated protein–protein interactions could be considered a putative noisy molecular model of the disease condition.

We used the Cytoscape plug-in, ActiveModules, to find an HSN for each condition. The plug-in employed a published algorithm [21], which consisted of a network scoring metric and a network search function. For a given network, the network score was computed as a standardized weighted average of the z-scores for the individual network nodes. The network search function was a simulated annealing algorithm that accounted for the effects of highly connected nodes (hubs) on the network score. Given the stated scoring metric and search function, the algorithm would find a subnetwork with a high score relative to its size. Because the network score was a weighted average of the z-scores of the individual member nodes, a network with a high score would be one where many of the individual members have relatively low *p*-values; i.e., the network, as a whole, would be differentially expressed in the disease condition (see Text S4 for additional details).

### Determining Gene-Set Enrichment in a Condition

To determine if a gene set, or equivalently, its functional subnetwork, was transcriptionally affected in a given condition, we tested if members of the gene set appeared disproportionately in the HSN for that condition, relative to the global network of protein–protein interactions. This was accomplished using Fisher's exact test, with a confidence level *α* = 0.05, and the gene set was considered enriched in the HSN if the *p*-value fell below *α*.

### Ranking Gene Sets by Number of Enriched Conditions

To identify the gene sets that were consistently deregulated across many DGAP conditions, each gene set was evaluated to see if its members were disproportionately present in many HSNs. The score of a gene set was defined as the number of HSNs in which it appeared enriched. Higher scores equated with enrichment of the gene set in more conditions, and the gene sets in the collection could be sorted and ranked based on their respective scores.

### Assigning Significance to Gene-Set Scores

A random walk approach on the protein–protein interaction network was used to determine the significance of a gene set's score. Specifically, 10,000 gene sets of the same size as the given set were generated by a random walk of the protein–protein interaction network (see Text S5 for details). Each random gene set was scored according to the method outlined above, and a distribution of all 10,000 scores was subsequently generated. The percentage of the random gene sets with scores equal to, or higher than, the given set was taken as the empirical *p*-value of the given set's score. *p*-Values <0.05 were considered significant. By comparing the gene set against random gene sets of the same size, the potential effects of the gene set's size on its score would be taken into account when determining the *p*-value.

Because HSNs were connected subnetworks, gene sets that formed dense functional subnetworks could potentially be identified as enriched in multiple HSNs by random chance. To determine potential biases towards dense networks and improve confidence in the identified gene sets, an additional analysis was performed on gene sets with significant *p*-values.

For each significant gene set, the densities for 10,000 random gene sets were determined in addition to their scores. Under the premise that dense networks were biased towards higher scores, the expectation would be to see a nondecreasing relationship between network densities and gene-set scores. A plot of network density versus gene-set score should then show such a relationship. If no such relationship was evident, then high network density could not have had a strong positive effect on the enrichment result.

### Software Availability

A documented distribution of the source code for the GNEA algorithm described in this paper is available from the authors upon request.

## Results

Impairment or alteration of the insulin-signaling pathway is a commonly recognized feature of type 2 diabetes. It is therefore notable that the IS-HD gene set (Dataset S4) was not detected to be significantly transcriptionally altered by application of either hypergeometric enrichmentt test, DEA or GSEA. In particular, applying GSEA to the transcriptional profile dataset of diabetic and normal glucose-tolerant skeletal muscle described in Mootha et al. [10] did not identify a significant level of alteration in the IS-HD gene set (*p* = 0.536), while DEA produced a comparably weak enrichment score (*p* = 0.607). The failure to detect a significant transcriptional alteration in IS-HD may be explained by a number of factors. The enrichment results depended on the specific choice of the IS-HD gene set, and it is possible that an alternatively defined insulin-signaling gene set would be determined as significantly enriched. Additionally, expression changes in a few critical genes in IS-HD may be sufficient to substantially alter insulin signaling, and running DEA on the large IS-HD set may miss the contributions from these few genes.

Interestingly, running GNEA on the dataset identified a significant alteration in the transcriptional profile of IS-HD. Because this particular analysis involved only a single dataset, the GNEA framework might not be strictly applicable and IS-HD was only assessed for overrepresentation in the HSN by Fisher's exact test and found to have a *p*-value of 0.034. To determine the robustness of the identified signature, 100 additional DM2 datasets were generated from the original using a bootstrap resampling technique [22]. Namely, each of the new datasets was generated by random sampling, with replacement, of individuals in the original. The mean *p*-value of the Fisher's exact test on the new datasets was 0.033 and the standard deviation was 0.092. To further improve our confidence in the result, a separate, nonrandom dataset was analyzed that compared between type 2 diabetic and normal individuals with no family history of type 2 diabetes [11]. IS-HD was also identified as transcriptionally altered in that dataset with a *p*-value of 0.026.

In addition, two lung cancer datasets [23,24] were also selected and analyzed using GNEA. A priori, identification of IS-HD in the two datasets would have reduced confidence in the relevance of the signature to DM2. IS-HD was, in fact, not identified in either of the two datasets, lending support to its possible relevance to the DM2 disorder.

The recurring signature of IS-HD alteration using bootstrap from a DM2 dataset and multiple insulin-resistance models in mouse improves our confidence in its relevance. The encouraging results motivated the larger study using model organisms described below and obviously require additional validation on a larger population of diabetic patients. It is also important to note that the positive GNEA result does not detract from the significance of DEA, GSEA, and related methods in other detection tasks (see results on glucose and fatty acid metabolism GO categories below).

**Figure 2:**
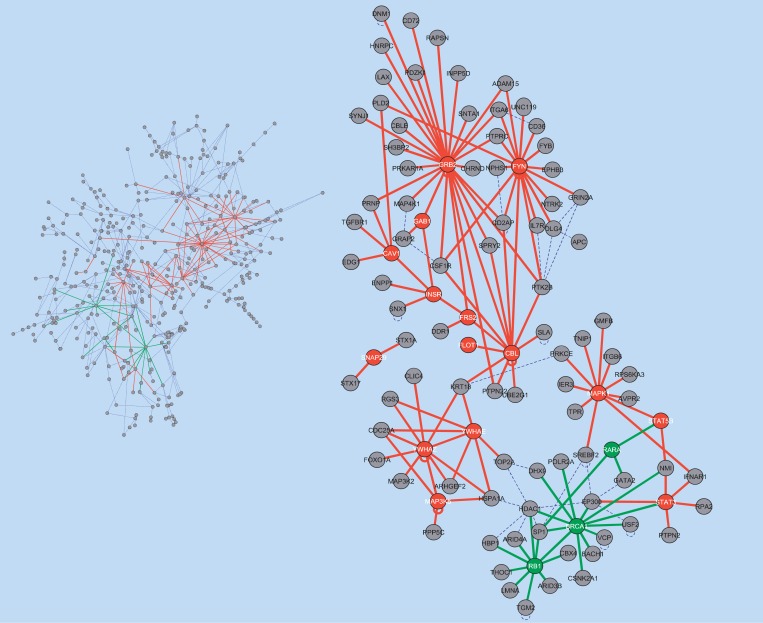
Comparison of Type 2 Diabetic and Normal Glucose Tolerant Skeletal Muscle (Left) The HSN is shown. Interactions involving at least one IS-HD member are highlighted in red. Those involving at least one NR-HD member are highlighted in green. Of the 417 nodes in the HSN, 102 belonged to or interacted directly with IS-HD or NR-HD members. (Right) The 102 nodes in the HSN formed a network signature. GNEA identified IS-HD, but not NR-HD, as enriched in this dataset. IS-HD and NR-HD members are highlighted in red and green, respectively.

The graphical result of the GNEA analysis on the skeletal muscle dataset is illustrated in Figure 2. As suggested intuitively by the figure and associated Fisher's exact test enrichment result (*p* = 0.034), the set of protein–protein interactions associated with the insulin-signaling pathway was significantly affected in diabetic skeletal muscle tissue compared to normal muscle tissue.

Motivated by the preliminary analysis of diabetic muscle tissue, we applied the GNEA methodology to the microarray gene-expression datasets compiled by the DGAP project. Insulin signaling, as represented by IS-HD, was found significantly transcriptionally altered in the largest number of insulin resistance and diabetes conditions compared to any other tested gene sets. The results confirm that insulin signaling is a strongly affected biological process in insulin resistance and diabetes. IS-HD was significantly affected across adipose, skeletal muscle, liver, and pancreatic tissue. While it may seem like a foregone conclusion to find changes in insulin signaling in insulin resistant conditions, the fact is that IS-HD was not identified as enriched in a significant number of conditions by DEA. Specifically, DEA found transcriptional alteration in IS-HD in only one condition comparing mouse liver tissue in high and low-fat diets. Insulin signaling is assumed to be deregulated in diabetes and insulin resistance phenotypes. The failure of DEA to find such deregulation in comparison to the success of GNEA underscores the importance of integration with network models (even in the form of protein–protein interactions) to enhance the ability to detect a biological signal.

**Figure 3:**
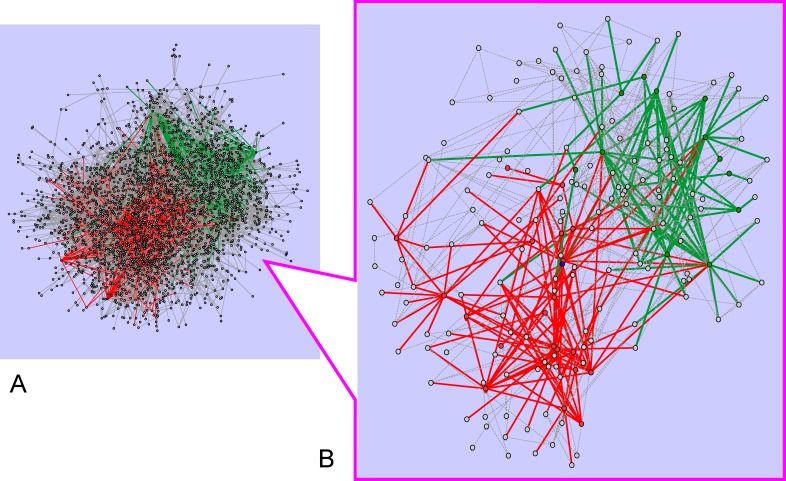
IS-HD and NR-HD Signaling (A) Protein–protein interactions appearing in at least one HSN are assembled together to form the network signature shown. Interactions involving at least one IS-HD member are in red. Those involving at least one NR-HD member are in green. (Interactions between IS-HD and NR-HD members are shown in green). (B) A subgraph of the same network that includes only the top 5% most frequently appearing genes in the HSNs is shown. Both IS-HD and NR-HD members clearly participate in numerous protein–protein interactions.

**Figure 4:**
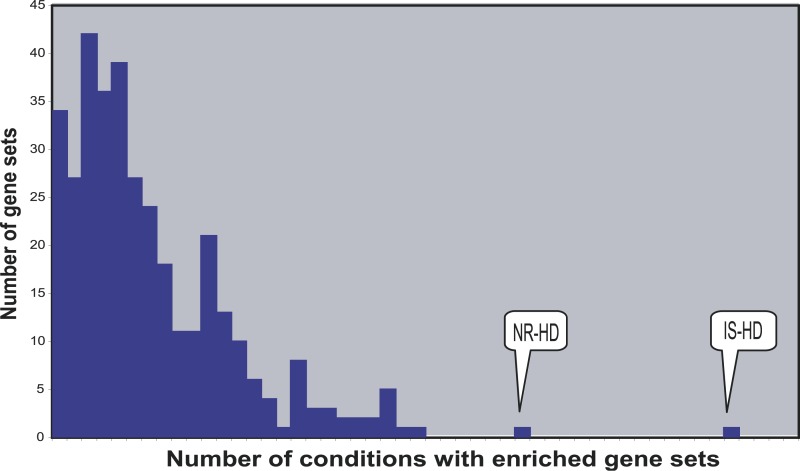
Frequency Histogram of Gene-Set Scores by GNEA This analysis used 353 gene sets, consisting of 346 GO categories, two retinol metabolism-signaling sets, three nuclear receptor sets, and the IS-HD and NR-HD gene sets. The indicated positions for the IS-HD (45) and NR-HD (31) gene sets correspond to the number of conditions where they were identified as enriched.

NR-HD was the second prominent gene set identified as significantly and consistently altered in diverse insulin-resistance models. Both IS-HD and NR-HD showed a widespread interactive role across different disease states (Figure 3), an abundance that was unmatched by any of the other tested gene sets (Figure 4). Furthermore, as suggested in Figures S2 and S3, the density of the gene sets in the underlying protein–protein interaction network did not appear to have had a strong effect on the scores for IS-HD and NR-HD.

Figure 4 summarizes the GNEA results for all tested gene sets and clearly demonstrates the distribution of the number of gene sets enriched in a given number of insulin resistance and diabetes conditions. The IS-HD and NR-HD sets were distinctly enriched in many more conditions than any other tested gene sets. The high enrichment of IS-HD may be explained by the central role of insulin signaling in insulin resistance phenotypes and the fact that many DGAP experiments specifically disrupted critical components of insulin signaling. The result for NR-HD requires further explanation. Individual nuclear receptors, such as HNF4A and PPARGs have been implicated in insulin resistance and type 2 diabetes in the past [13–17]. More generally, many nuclear receptors are known to form monomeric, heterodimeric, or homodimeric complexes and act to regulate diverse metabolic processes [25]. Because the NR-HD set was dominated by nuclear receptors and their cofactors, it is possible that the high enrichment of the set was a consequence of the key metabolic roles played by individual nuclear receptors, coupled with their interactions with cofactors and other partners. Under this scenario, the NR-HD set may be viewed as a compilation of key players involved in the metabolic distress phenotypes associated with DM2.

Intriguingly, when the genes in NR-HD were ranked by the number of times they appeared in all the HSNs, no individual gene appeared more than 18 times. Given that the NR-HD set was enriched in 31 conditions, this suggested that different subsets of NR-HD were responsible for the enrichment in different conditions. To investigate whether a recurring subnetwork of NR-HD was responsible for enrichment in multiple conditions, we computed a Pearson correlation coefficient for every pair of genes in NR-HD based on their binary profile of appearances in the 31 enriched HSNs. While some genes tended to cluster, no group of genes stood out as strongly correlated in these profiles (Figure S4). The results suggested that different insulin resistance conditions were associated with deregulation of different subsets of NR-HD. This scenario illustrates an advantage of the GNEA approach over more conventional ones such as DEA. While the latter may identify enrichment of individual NR-HD members, it would fail to find enrichment of the set as a whole since no gene appeared enriched in as many conditions as the total set.

The NR-HD set was also identified as significantly transcriptionally altered by DEA. However, DEA identified NR-HD enriched in far fewer conditions than GNEA (six compared to 31). Interestingly, the three other nuclear receptor gene sets were not identified as enriched in a significant number of conditions by either DEA or GNEA. The lack of enrichment in the nuclear receptor superfamily gene set can be explained by the different patterns of expression of individual nuclear receptors in diverse tissues [26]. However, the similar results for the HUGO nuclear receptor gene set, and the union of these two gene sets, supported a broader conclusion. Since none of the three nuclear receptor gene sets were enriched in a significant number of conditions while NR-HD was, the results suggested that only a specific subset of nuclear receptors (including the NR-HD genes) and their constellated cofactors was differentially associated with insulin resistance and DM2.

### NR-HD Was Enriched in Many DGAP Conditions

**Figure 5:**
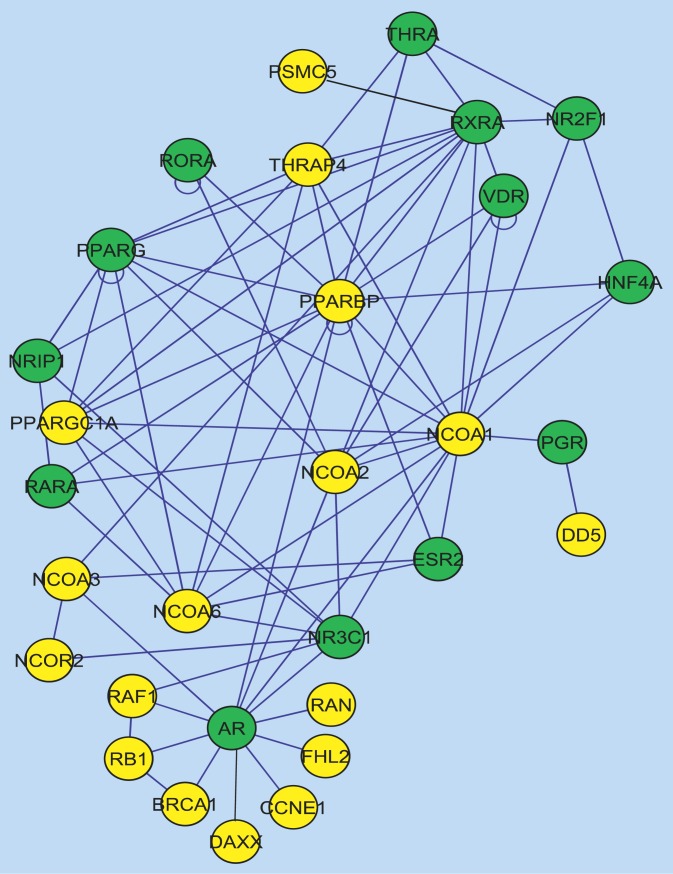
The NR-HD Gene Set The NR-HD gene set was composed of 13 nuclear receptors (green) and 17 nuclear receptor coregulators (gold). Black edges denote interactions documented in the most recent HPRD version that were not well documented in the version used in the analysis.

**Figure 6:**
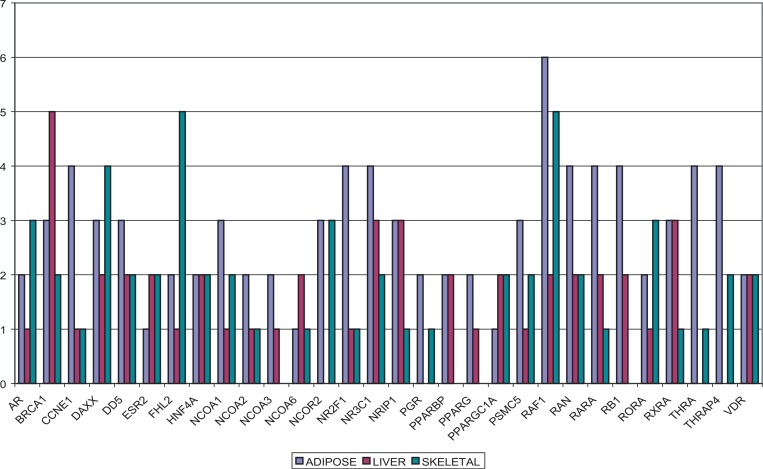
The Distribution of NR-HD Gene Memberships in the 31 HSNs The height of the bars reflects the number of HSNs in which a NR-HD gene appeared. NR-HD was enriched in 13, nine, and nine conditions associated with adipose, liver, and skeletal muscle, respectively.

The NR-HD set (Figure 5) was found enriched in 31 conditions by GNEA (raw *p* = 0.0030 and false discovery rate = 0.0179) and in six by DEA (*p* = 0.0125, false discovery rate = 0.0875). The two methods found six conditions in common where NR-HD was enriched. All of the found conditions from GNEA or DEA corresponded to comparisons between an insulin resistant or diabetic state and normal. The 31 conditions spanned 13 adipose, nine liver, and nine skeletal muscle tissues. The six conditions spanned three adipose, two liver, and one skeletal muscle tissue. Some genes in NR-HD had well-documented associations with DM2, such as *PPARG, PPARGC1A,* and *HNF4A* [10,13–17]. Other genes from the NR-HD set could also be connected to DM2 via their associations with diabetes risk factors, including *glucocorticoid receptor* (*GR,* also known as *NR3C1)* [27] and *vitamin D receptor (VDR)* [28]. While these genes had been individually associated with insulin resistance and DM2 in the past, to our knowledge, this was the first time a set of nuclear receptors had been identified as significantly transcriptionally altered in multiple insulin resistance and diabetes models. The set also included genes related to retinol metabolism and signaling, such as *retinoic-acid receptor alpha (RARA), retinoid X receptor alpha (RXRA),* and *retinoic-acid receptor-related orphan receptor alpha (RORA)*. Previous studies had suggested a possible connection between retinol metabolism signaling and DM2 [29–31]. As illustrated in Figure 5, 13 NR-HD genes were nuclear receptors, some of which acted as mediators for diverse metabolic processes [25,32]. The remaining 17 NR-HD genes were nuclear receptor coregulators. The distribution of gene memberships in the 31 HSNs found enriched by GNEA was examined and grouped by tissue type, with the results shown in Figure 6. *v-raf-1 murine leukemia viral oncogene homolog 1 (RAF1)* was present in the most number of HSNs, appearing in 13 of the 31 conditions and all three tissue types. GR was the most frequently appearing nuclear receptor, present in nine of the conditions and also all three tissue types. Certain genes tended towards specific tissue types: *four and a half LIM domains 2 (FHL2)* appeared in eight conditions overall, five of which were skeletal muscle. *Nuclear receptor subfamily 2, group F, member 1 (NR2F1)* appeared in six conditions overall, four of which were adipose.

### IS-HD Was Enriched in Many DGAP Conditions

**Figure 7:**
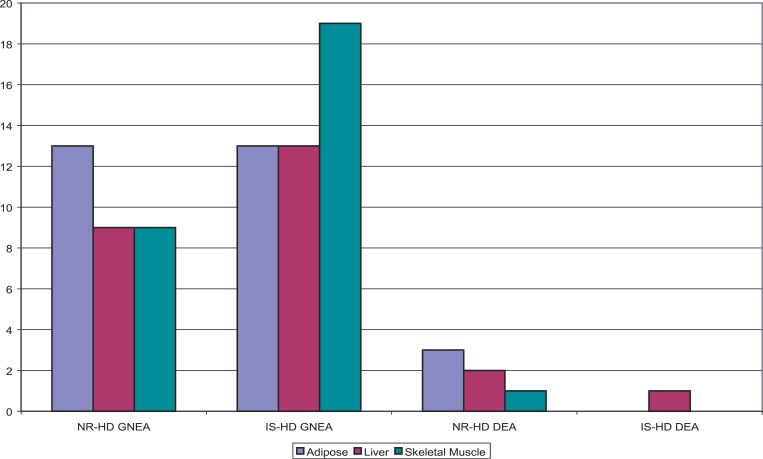
GNEA and DEA Enrichment Results for Both NR-HD and IS-HD NR-HD was enriched in 31 and six conditions by GNEA and DEA, respectively, and IS-HD was enriched in 45 and one. The distribution of enrichments in adipose, liver, and skeletal muscle tissue is shown. IS-HD was additionally enriched in one DGAP condition in pancreatic tissue by GNEA (not shown).

The IS-HD set was found enriched in 45 conditions by GNEA (raw *p* = 0.0050, false discovery rate = 0.0179) that compared between an insulin resistant or diabetic state and normal. The conditions spanned diverse tissues (13 adipose, 13 liver, 18 skeletal muscle, and one pancreas tissue) and suggested a widespread tissue independent effect. When the genes in IS-HD were ranked by the number of times they appeared in the 67 HSNs, no individual gene appeared more than 18 times. This result suggested that, similar to NR-HD, different components of IS-HD were being deregulated in different insulin resistance models. This is consistent with the fact that multiple deregulatory models exist for insulin resistance [33,34]. Figure 7 summarizes the results for NR-HD and IS-HD under both GNEA and DEA.

### Overlap between NR-HD and IS-HD

**Figure 8:**
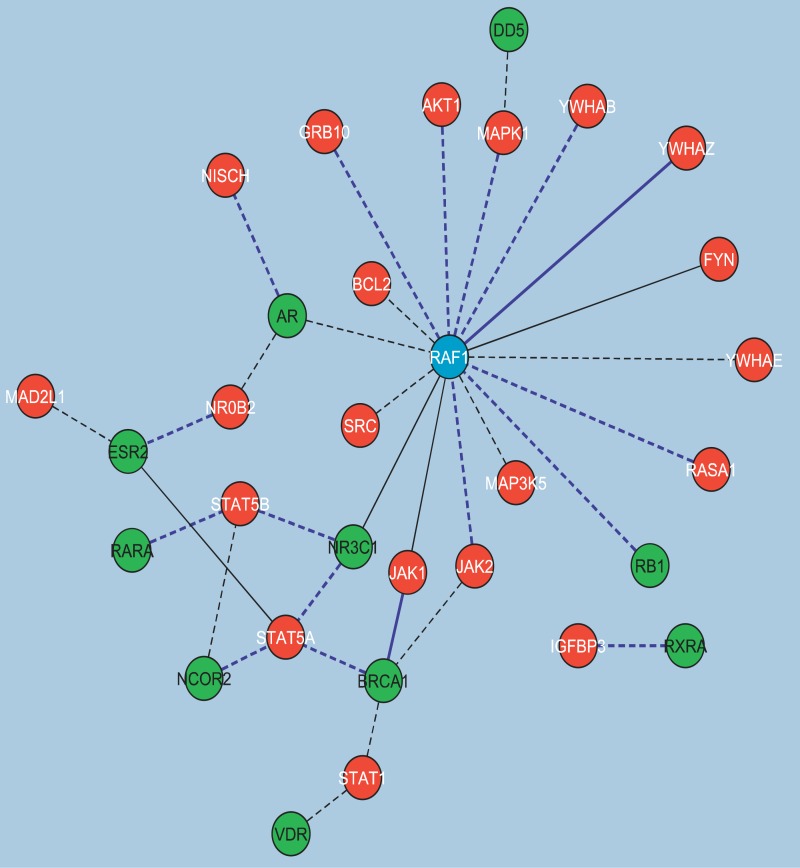
Protein–Protein Interactions between NR-HD and IS-HD The protein–protein interactions between members of NR-HD (in green) and IS-HD (in red) in the conditions where both sets appear enriched form a mostly connected network. RAF1 (in blue) was a member of both sets. Of the 33 total interactions, 16 involved RAF1. Interactions shown in dotted black, solid black, dotted blue, and solid blue appeared in one, two, three, and four HSNs, respectively.

GNEA identified IS-HD significantly enriched in 45 conditions and NR-HD in 31. Both were identified enriched in 22 conditions spanning nine adipose, seven liver, and six skeletal muscle tissue. One explanation for the overlap is that certain genes may act as signaling mediators or serve dual functions between the two gene sets. Transcriptional alteration in one set may therefore affect the other. To pursue this hypothesis, protein–protein interactions between IS-HD and NR-HD were identified and examined. The interactions were observed to form a connected subnetwork (Figure 8). IS-HD genes involved in these interactions included the *signal transducer and activator of transcription family (STAT5A, STAT5B,* and *STAT1), janus kinases (JAK1* and *JAK2),* and *tyrosine 3-monooxygenase/tryptophan 5-monooxygenase activation proteins*, *(YWHAB, YWHAZ,* and *YWHAE)*. NR-HD genes included *androgen receptor (AR), breast cancer 1 (BRCA1),* and *GR (*also known as *NR3C1)* among others. Interestingly, *RAF1,* which participated in the most interactions between the two gene sets, was also a member of both. Possibly, among other potential signal transductions, insulin deregulation may propagate a signal through *RAF1* to the nuclear receptors.

### GNEA and DEA: Side-by-Side Comparison

As previously mentioned, GNEA found IS-HD and NR-HD enriched in 45 and 31 conditions, respectively, whereas DEA found enrichment in only one and six. In addition, we systematically compared the results from GNEA and DEA on the two retinol signaling gene sets and three nuclear receptor gene sets, as well as the group of 346 GO category gene sets present in the DGAP expression data. None of the retinol signaling or nuclear receptor gene sets appeared enriched in a significant number of conditions by either method. Top-scoring GO categories in GNEA included RNA splicing, cell-growth/death regulation, and nuclear receptor signaling processes with the latter category explained by the strong enrichment of the NR-HD set. Top ranked GO categories in DEA include metabolism and cell-growth/death-regulation processes. Growth is intimately tied to metabolism, and the appearance of such categories in both GNEA and DEA may be explained by the deregulation of the latter. Moreover, certain nuclear receptors had been associated with apoptosis pathways and cancer in the past [35,36], and the top scores of cell-growth/death-regulation GO categories were likely partly due to the general enrichment of the NR-HD set members.

Excluding IS-HD and NR-HD, no tested, potentially disease-associated gene set scored significantly by GNEA, nor did any GO category gene sets. Interestingly, a number of metabolism-related GO categories scored significantly by DEA. Because insulin resistance is associated with dysregulation of metabolism, the expectation was to see a significant score for such GO categories. DEA results confirmed the expectation whereas GNEA did not. In particular, GO categories such as GO:0006006 (glucose metabolism) and GO:0006631 (fatty acid metabolism) had very low scores in GNEA and demonstrated a caveat with the network-based approach. The low gene-set scores resulted from the very disconnected functional subnetworks for these gene sets. Because HSNs were connected subnetworks, gene sets with connected functional subnetworks tended to be better represented in an HSN than those without. By implication, biological processes that were poorly represented by protein interactions, such as oxidative phosphorylation, would be missed by GNEA. While an acknowledged shortcoming, we note that disconnected gene sets in the present will become increasingly connected in the future as knowledge about them accumulates with time and as the interactions of member genes are cataloged by the on-going efforts of projects such as the HPRD [37]. At present, the GNEA methodology provably succeeded at identifying two gene sets, NR-HD and IS-HD, which were clearly associated with insulin resistance models despite this caveat. Hence, its utility is clear even with the presently available information.

### Little Expression Correlation among Insulin Resistance Models

**Figure 9:**
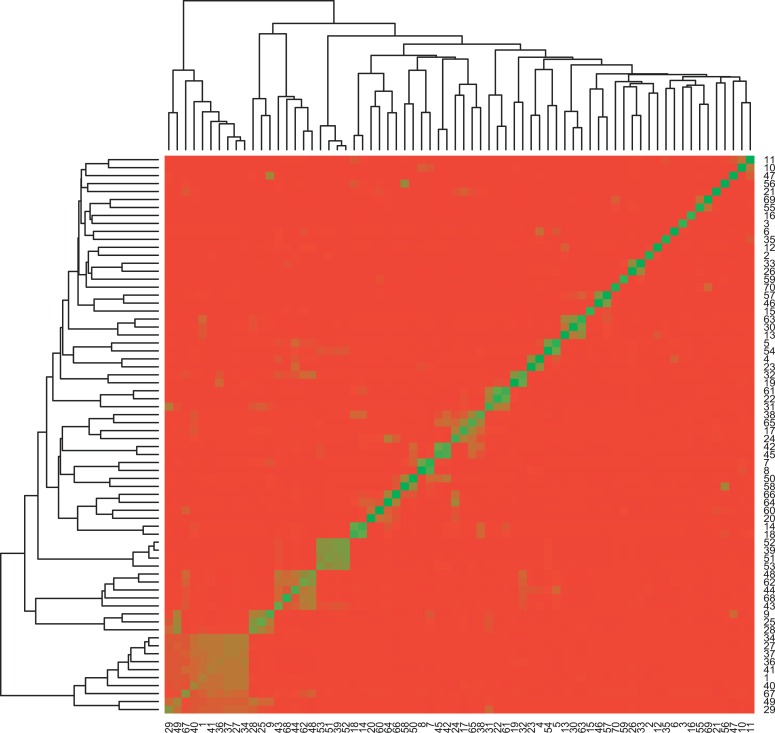
Pearson Correlation Coefficients between Every Pair of DGAP Conditions The gene-expression profiles for the DGAP conditions, (indexed by their numerical identifiers, Dataset S3), were generally poorly correlated.

We computed a Pearson correlation coefficient between every pair of DGAP conditions based on their global gene expression (Figure 9). The results suggested reasonably little gene-expression correlation between different insulin resistant models. The lack of clustering reinforced the observation that different sets of genes and cause-effect mechanisms were responsible for the insulin resistance phenotype in different models.

## Discussion

In its most ambitious form, system biology aims to understand the molecular mechanisms of normal and disease states and develop a better understanding of genetic and environmental factors, including drugs, affecting these mechanisms in different conditions. System level approaches have been generating increasingly deeper insights in areas where the combination of current knowledge and measurement technologies provide a sufficiently accurate and interpretable snapshot of biological processes [38–41]. In particular, integration of genome-wide measurements and protein–protein interaction networks has produced a number of functional and regulatory predictions that have been validated in biological assays. We follow this integrative approach by introducing GNEA, a simple but promising strategy for identifying transcriptionally altered processes in a set of abnormal biological states caused by disease or pharmacological interventions. GNEA differs from traditional enrichment approaches in a number of ways. First, it makes use of protein–protein interaction information. Second, it examines transcriptionally altered sets of genes rather than individual genes. Finally, it integrates gene-expression datasets from diverse disease models. Each of these distinctions confers an advantage to GNEA over widely used methods such as DEA as well as some potential caveats.

By organizing genes into a network based on the physical interactions among their protein products, GNEA imposes constraints on the relationships between genes. It has previously been demonstrated that, with the exception of protein complexes, genes with interacting protein products do not necessarily have well-correlated gene-expression profiles [42]. Protein–protein interactions therefore represent a nonredundant line of evidence that can be combined with gene-expression information to elucidate the interrelationships between genes and their participation in biological processes. For biological processes with well-characterized protein interactions, such as insulin signaling, GNEA demonstrably identifies those that are altered in disease phenotypes. However, it is less effective in situations where the biological processes are poorly characterized in terms of protein–protein interactions or where such information is potentially inaccurate. In the former, GNEA may fail to find enrichment of a gene set because the interaction information is insufficiently comprehensive and the gene-set members are highly disconnected from one another as a result. In the latter, GNEA may give an inaccurate result because the protein–protein interaction information itself contains inaccuracies. Considering its dependence on reliable protein–protein interaction information, GNEA is best viewed as a complement to existing methods, such as DEA and GSEA [18], which do not rely on such information. For situations where the protein–protein interaction information is comprehensive, the results from GNEA are likely more sensitive. When protein–protein interaction information is lacking, such results may be considered subordinate to those from the other methods.

GNEA tests for transcriptional alteration in sets of genes rather than individual genes. Under this approach, individual genes are not required to be differentially expressed in order to identify biological processes or gene sets that are cumulatively differentially expressed. Some genes, such as transcription factors, play important roles in biological processes without necessarily showing a large change in transcriptional activity when the processes are altered. Gene sets that contain many such genes would be missed under the criterion that individual genes be differentially expressed. GNEA provides an alternative where a gene's expression value and its interactions with other genes are both taken into consideration. In this way, genes that are not individually significant may still be taken into account if they are connected to other significant genes. As examples, both IS-HD and NR-HD contained individually nonsignificant genes despite being identified as cumulatively differentially expressed sets (Dataset S5). In the converse situation, a dataset may contain thousands of differentially expressed genes, in which case numerous gene sets would be overrepresented in differentially expressed genes. GNEA also applies in this instance by favoring those gene sets with well-connected members in the protein–protein interaction network. The downside to such an approach is that, in common with all gene-set enrichment approaches, the enrichment result for any gene set will depend on its composition. For a given biological process, two different, yet equally representative, sets of genes may happen to show different enrichment results. We emphasize, however, that while the composition of the gene sets may affect the enrichment results, the sizes of the gene sets do not. As previously described in the Methods section, GNEA explicitly compares the performance of each test gene set against those from random gene sets of the same size. Biases resulting from the sizes of the test gene sets are therefore taken into account when determining the significance values.

The third distinction between GNEA and existing methods is its focus on identifying biological signals that are recurrent across multiple, rather than individual, conditions. While such a focus allows GNEA to pick up biological signals that might otherwise be missed in any one condition individually, it also means that the analysis is influenced by the choice and number of conditions being tested. Some biological processes of interest may be enriched in only a specific subset of the total conditions being tested. Such processes may be missed when the total number of tested conditions is large. The signal is effectively diluted because the conditions are too removed from the context where the process is active. Selecting the proper and relevant conditions for testing must therefore be done carefully. The converse situation is also possible. When the number of tested conditions is low, even a gene set that is enriched in all conditions may nonetheless have an insignificant *p*-value, as the probability of such an observation occurring by random chance is high. In such instances, one potential workaround is to employ a bootstrap approach similar to the one described for the validation of the IS-HD enrichment signal in the DM2 dataset (see Results).

Finally, it should be noted that the effect of constraining genes by protein–protein interactions and then testing for gene-set enrichment by GNEA is different from the effect of merely lowering the significance threshold and testing for gene-set enrichment by DEA. In general, not all genes in a dataset are connected to each other through protein–protein interactions. Consequently, the genes taken into consideration by GNEA would be different from the ones determined by significant value alone, independent of the significance level used. Because the connection between genes by protein interactions is independent of the significance level, GNEA is potentially less dependent on the threshold used to identify differentially expressed genes compared to conventional methods such as DEA [43].

Our work shares the philosophy advocated in He and Zhang [44]. There, the authors argued that the reason many hubs in protein–protein interaction networks correspond to essential proteins is that hubs have a higher probability of involvement in essential protein–protein interactions. This paradigm, of identifying variables correlating with key protein–protein interactions, might be generalized to phenotypes such as insulin resistance. That is, the identification of processes with relatively many protein–protein interactions essential for proper insulin sensitivity might assist the identification of those affected in insulin resistance.

GNEA is not the first method to integrate gene-expression data with protein–protein interaction information for various analytical tasks. For example, Steffen et al. [45] and Ideker et al. [46] combined gene-expression data with protein–protein interaction networks to identify signal transduction pathways in yeast. Others, such as Karaoz et al. [47], attempted to improve functional gene classification. In contrast, GNEA aims to detect biological processes that are consistently altered across multiple disease models. Gene-expression measurements are integrated with protein–protein interaction information to identify transcriptionally affected subnetworks that can be considered as noisy molecular signatures of specific disease conditions. Biological processes that are overrepresented in a significant number of disease models are identified as deregulated in the phenotype.

Using GNEA, our results indicated that the insulin-signaling process is significantly transcriptionally altered in insulin resistance and DM2. While this observation appears quite natural, it remains that other techniques have difficulty detecting such a deregulation. The alteration in insulin signaling was identified across adipose, liver, skeletal muscle, and pancreas tissues. Insulin signaling is well associated with DM2, and the relationship between impairment in this biological process and insulin resistance has been supported by numerous clinical investigations and genetic studies. The results likewise implicated a specific set, NR-HD, of nuclear receptors whose individual members are known to be involved in diverse sensory and transcriptional processes and have been previously associated with insulin resistance and DM2. The nuclear receptor set includes *HNF4A, PPARG, PPARGC1A,* and other genes that have been implicated with diabetes in previous association and linkage studies [10,27,48–50]. The association of NR-HD with changes in transcriptional activation is somewhat surprising as nuclear receptors are ligand activated. Further studies will be needed to examine the roles played by the NR-HD genes, their relationships to each other, and the nature of their association, as a set, with DM2 and insulin resistance models. IS-HD and NR-HD members were shown to interact in a mostly connected, protein–protein interaction network. Moreover, *RAF1* was demonstrated to be a hub in many of these interactions, across a variety of tissue types, and may be an important signaling mediator between the two processes. In addition to *RAF1,* other NR-HD genes were also observed to be protein–protein interaction hubs.

More generally, the results for the insulin signaling and nuclear receptor sets suggest that different members of these sets were transcriptionally altered under different insulin-resistance models. Different insulin-resistance models also showed little correlation in gene expression with each other. While these results might be a consequence of biological or experimental noise common to microarray studies, they are also consistent with the notion of a combinatorial disease model whereby deregulation of different and small sets of genes may independently lead to the insulin resistance phenotype.

## Supporting Information

Dataset S1 Experimental Description for Each of the 16 DGAP Datasets This table gives a brief description of each of the DGAP datasets used in the analysis. Full experimental details are available on the DGAP website (http://www.diabetesgenome.org). The first column lists the dataset identifier (16 total, identifiers “3” and “9” are absent). The second column iterates the Affymetrix microarrays used in each dataset. The third and final column gives a brief description of the experimental design in each dataset.
(15 KB XLS)

Dataset S2 Information on Each of the 361 Microarrays This table gives a brief description of each of the microarrays used in the analysis. Full experimental details are available on the DGAP website. The first column lists the dataset (indexed by dataset identifier) from which the model was taken. The second and third columns iterate the Affymetrix microarray type and number of them used to profile the model. The fourth through seventh columns give a short description, species, sampled tissue type, and expected genotype of the model, respectively. The final column gives a fuller description of the model.
(48 KB XLS)

Dataset S3 Information on Each of the 67 Conditions This table details the comparison between the perturbed state and normal for each of the DGAP conditions. The first column gives the condition identifier for each model. Models with the same condition identifiers are compared to each other in the specified condition. It is important to note that the condition identifiers run from 1 to 54, and then from 56 to 68. Condition number 55 was not analyzed because the models being compared in it contained mislabeled data. The second and third columns list a short description and sampled tissue type of each of the models being compared.
(26 KB XLS)

Dataset S4 The IS-HD Gene Set and Protein–Protein Interactions This table includes all members in the IS-HD gene set and lists all edges in the IS-HD functional subnetwork. Gene members are referenced by their National Center for Biotechnology (http://www.ncbi.nlm.nih.gov) gene symbol. The genes in the first column share an edge with those in the second. Genes without a partner in the second column are disconnected in the functional subnetwork.
(17 KB XLS)

Dataset S5 IS-HD and NR-HD Gene Significance Values in the 67 Conditions This table contains the significance values of all genes in IS-HD and NR-HD for each of the DGAP conditions, prior to multiple-testing correction. The first four columns list the human gene symbols, Entrez Gene databank identification numbers, membership in IS-HD, and membership in NR-HD. The remaining 67 columns, indexed by condition identifiers, give the significance values of the genes in each condition.
(165 KB XLS)

Figure S1 Overview of DEA (1) A collection of gene sets associated with biological processes was assembled. (2) Each gene set was tested for overrepresentation in differentially expressed genes (*p* < 0.05) based on a Fisher's exact test. A gene set was considered enriched in the condition if the Fisher's *p*-value fell below 0.05. (3) We repeat step 2 for each condition. (4) Gene sets were ordered based on the number of conditions in which they are enriched. (5) For each gene set, the number of conditions in which it is enriched was assigned a *p*-value based on comparison against a background distribution.
(465 KB PDF)

Figure S2 Scatter-Plot of Network Density versus Gene-set Score for NR-HD The network densities for 10,000 random gene sets of the same size as NR-HD were plotted versus their gene-set scores. Network density did not appear to have had a strong, positive effect on gene-set score.
(894 KB PDF)

Figure S3 Scatter-Plot of Network Density versus Gene-Set Score for IS-HD The network densities for 10,000 random gene sets of the same size as IS-HD were plotted versus their gene-set scores. Network density did not appear to have had a strong positive effect on gene-set score.
(946 KB PDF)

Figure S4 Pearson Correlation Coefficients between NR-HD Genes Each NR-HD gene was associated with a binary profile of appearances in the 31 HSNs where NR-HD was enriched. The Pearson correlation coefficient was computed for every pair of genes in NR-HD based on their binary profiles. No group of genes stood out as strongly correlated in these profiles.
(311 KB PDF)

Text S1 Compilation of the Gene-Expression Data (25 KB DOC)

Text S2 Assembly of the Gene Sets (35 KB DOC)

Text S3 Assembly of the General and Condition-Specific Interaction Networks (24 KB DOC)

Text S4 Cytoscape Algorithm and Running Parameters (20 KB DOC)

Text S5 Procedure to Generate Random Gene Sets in GNEA and DEA (20 KB DOC)

### Accession Numbers

The Entrez Gene databank (http://www.ncbi.nlm.nih.gov/entrez) accession numbers for the genes discussed in this paper are *AR,* 367; *BRCA1*, 672. ; *FHL2,* 2274; *GR,* 2908; *HNF4A,* 3172; *JAK1,* 3716; *JAK2,* 3717; *NR2F1,* 7025; *PGC1A,* 10891; *PPARG,* 5468; *RAF1,* 5894; *RARA,* 5914; *RORA,* 6095; *RXRA,* 6256; *STAT1,* 6772; *STAT5A,* 6776; *STAT5B,* 6777; *VDR,* 7421; *YWHAB,* 7529; *YWHAE,* 7531; and *YWHAZ,* 7534.

Entrez Gene databank accession numbers for all additional genes in the IS-HD and NR-HD gene sets can be found in Dataset S5.

## Abbreviations

DEA: hypergeometric enrichment test on differentially expressed genes
DGAP: Diabetes Genome Anatomy Project
DM2: type 2 diabetes mellitus
GNEA: gene network enrichment analysis
GO: gene ontology
GSEA: gene-set enrichment analysis
HNF4A: hepatocyte nuclear factor 4 alpha 1
HPRD: Human Protein Reference Database
HSN: high-scoring subnetwork
IS-HD: insulin-signaling gene set used in the analysis of the DGAP dataset and the HPRD protein–protein interactions
NR-HD: nuclear receptor signaling gene set used in the analysis of the DGAP dataset and the HPRD protein–protein interactions
