# Supplementary material for: Network-Based Analysis of Affected Biological Processes in Type 2 Diabetes Models
Source: PLoS Genet. 2007 Jun 15;3(6):e96. doi: 10.1371/journal.pgen.0030096 (PMC1904360; doi:10.1371/journal.pgen.0030096)
Supplement: Supplementary file 1 [file pgen.0030096.sg001.pdf]

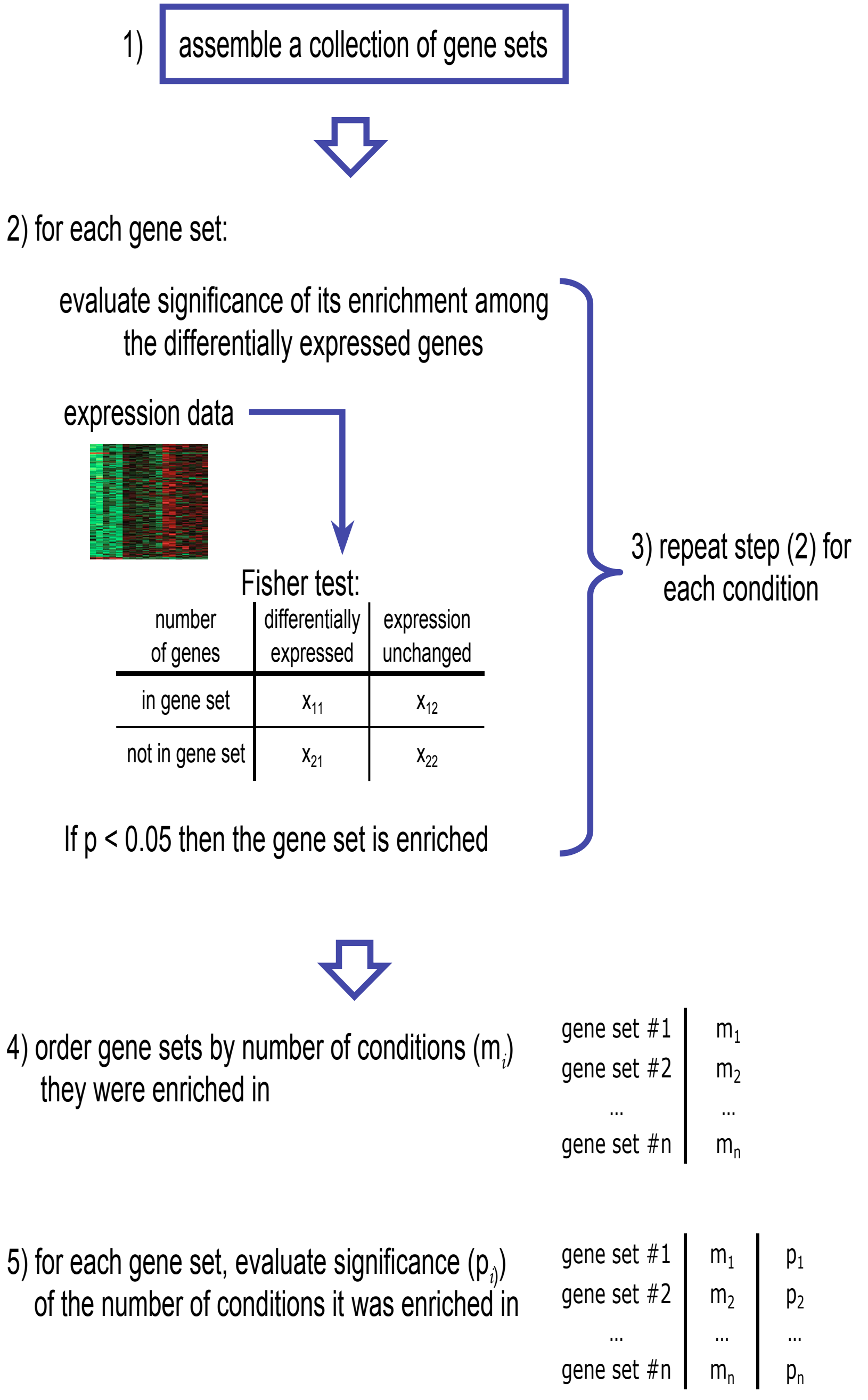
1) assemble a collection of gene sets
2) for each gene set:
evaluate significance of its enrichment among the differentially expressed genes
expression data
Fisher test:
number of genes
differentially expressed
expression unchanged
in gene set
x_{11}
x_{12}
not in gene set
x_{21}
x_{22}
If $p < 0.05$ then the gene set is enriched
3) repeat step (2) for each condition
4) order gene sets by number of conditions (m_i) they were enriched in
gene set #1 | m_1
gene set #2 | m_2
... | ...
gene set #n | m_n
5) for each gene set, evaluate significance (p_i) of the number of conditions it was enriched in
gene set #1 | m_1 | p_1
gene set #2 | m_2 | p_2
... | ... | ...
gene set #n | m_n | p_n
