# Supplementary material for: Network-Based Analysis of Affected Biological Processes in Type 2 Diabetes Models
Source: PLoS Genet. 2007 Jun 15;3(6):e96. doi: 10.1371/journal.pgen.0030096 (PMC1904360; doi:10.1371/journal.pgen.0030096)
Supplement: Supplementary file 2 [file pgen.0030096.sg002.pdf]

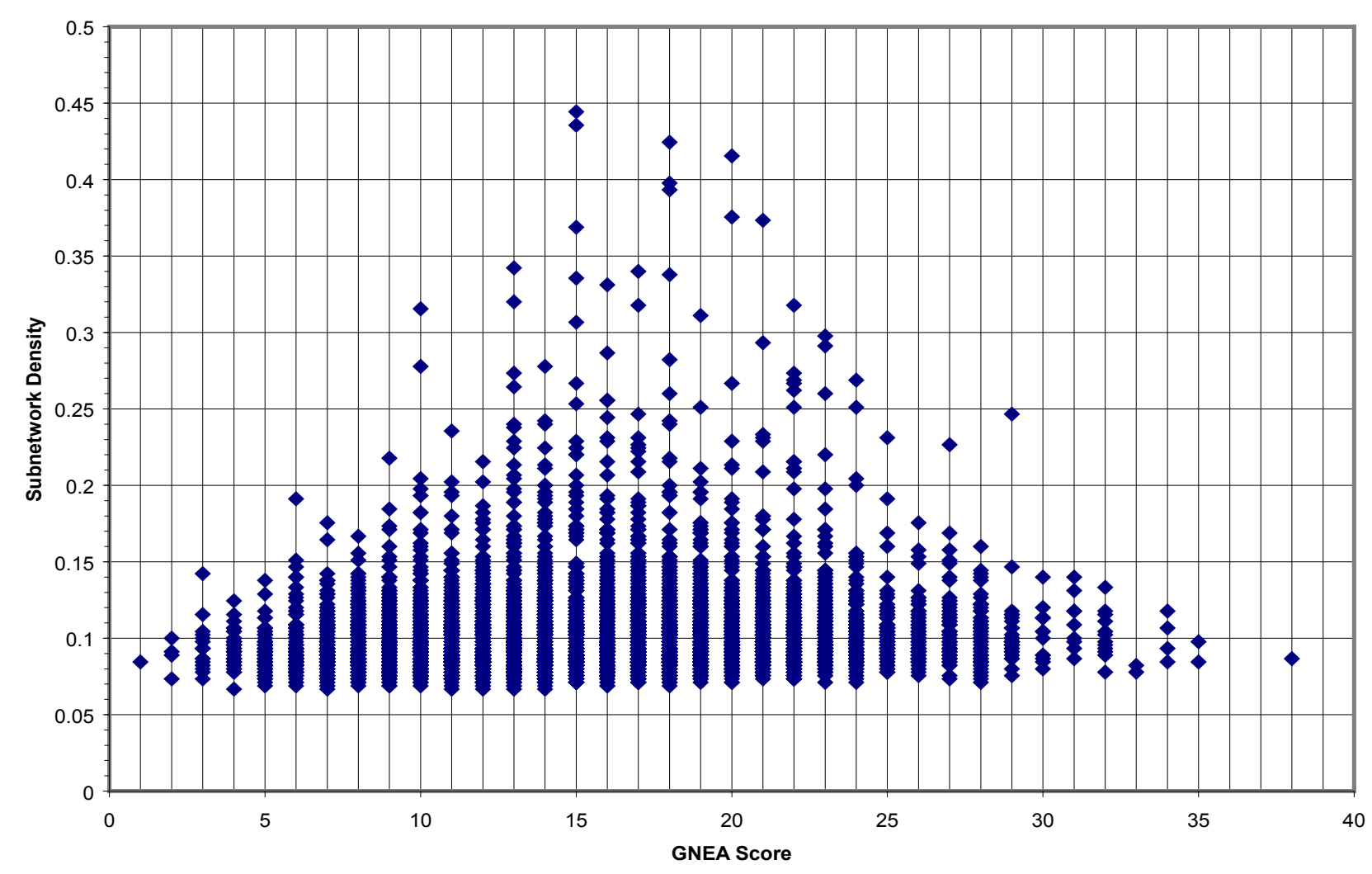
Subnetwork Density
GNEA Score
0.5
0.45
0.4
0.35
0.3
0.25
0.2
0.15
0.1
0.05
0
0
5
10
15
20
25
30
35
40
