# Supplementary material for: Network-Based Analysis of Affected Biological Processes in Type 2 Diabetes Models
Source: PLoS Genet. 2007 Jun 15;3(6):e96. doi: 10.1371/journal.pgen.0030096 (PMC1904360; doi:10.1371/journal.pgen.0030096)
Supplement: Supplementary file 3 [file pgen.0030096.sg003.pdf]

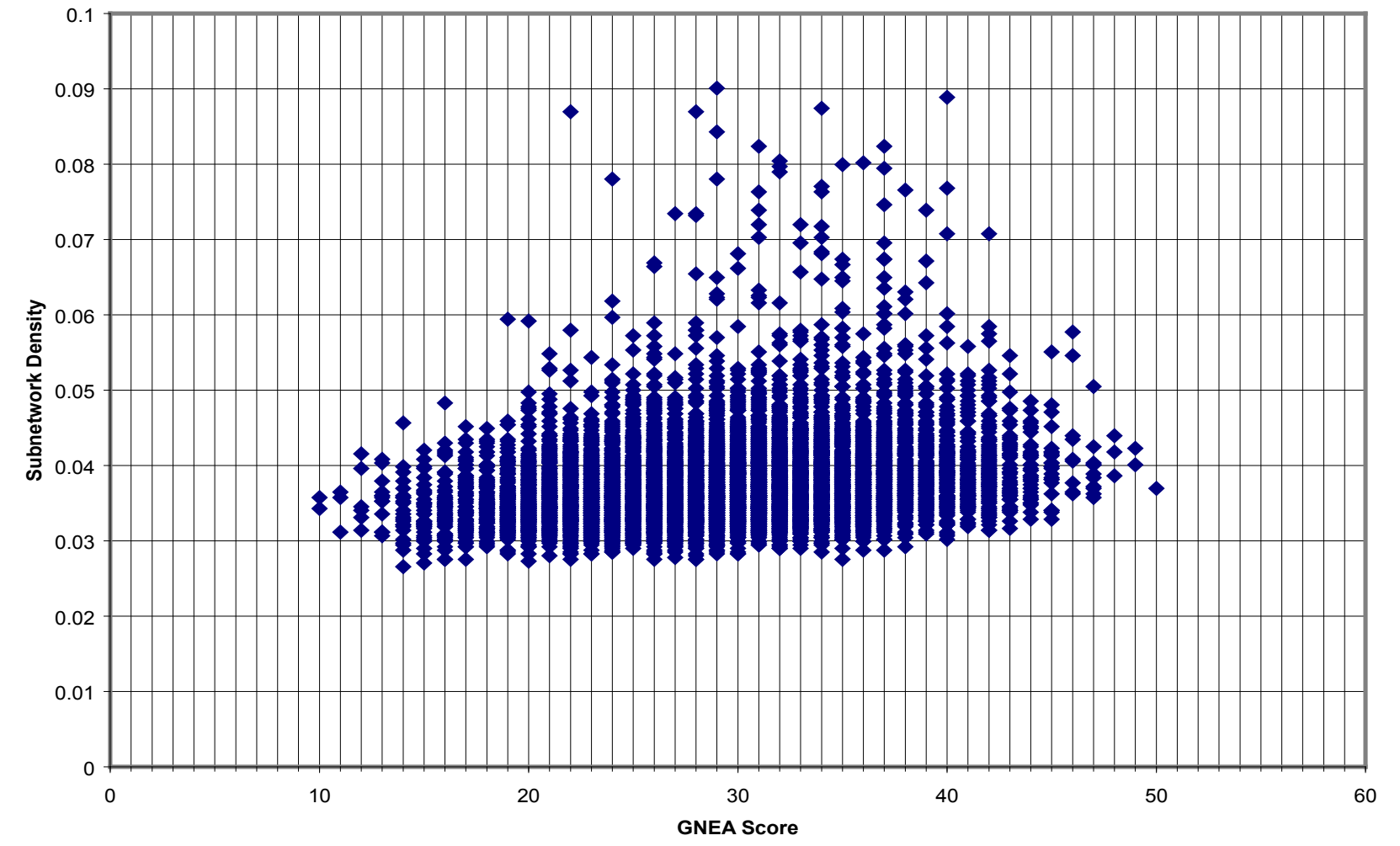

Subnetwork Density
0.1
0.09
0.08
0.07
0.06
0.05
0.04
0.03
0.02
0.01
0
0
10
20
30
40
50
60
GNEA Score
