# Supplementary material for: Network-Based Analysis of Affected Biological Processes in Type 2 Diabetes Models
Source: PLoS Genet. 2007 Jun 15;3(6):e96. doi: 10.1371/journal.pgen.0030096 (PMC1904360; doi:10.1371/journal.pgen.0030096)
Supplement: Supplementary file 4 [file pgen.0030096.sg004.pdf]

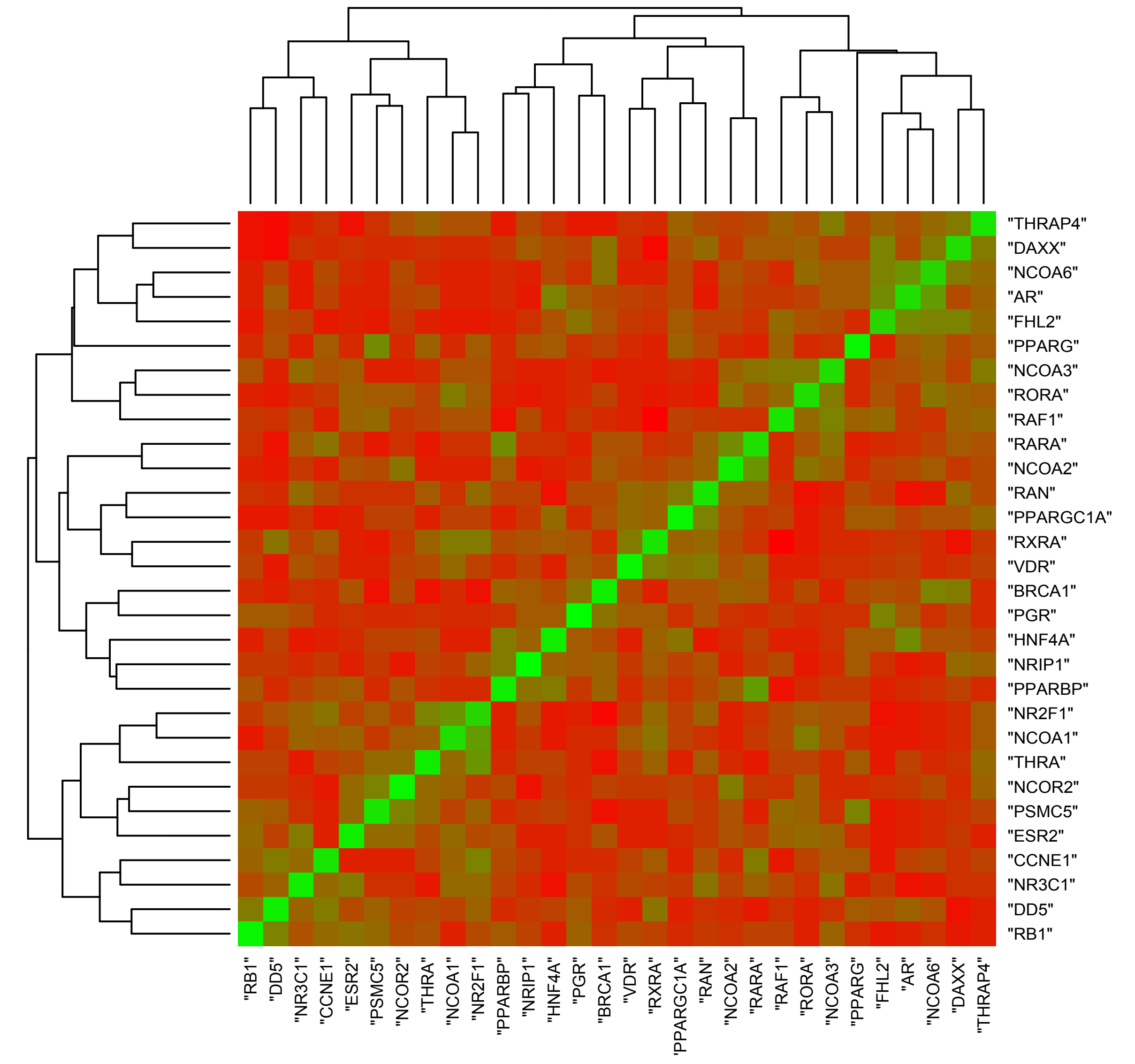

"THRAP4"
"DAXX"
"NCOA6"
"AR"
"FHL2"
"PPARG"
"NCOA3"
"RORA"
"RAF1"
"RARA"
"NCOA2"
"RAN"
"PPARGC1A"
"RXRA"
"VDR"
"BRCA1"
"PGR"
"HNF4A"
"NRIP1"
"PPARBP"
"NR2F1"
"NCOA1"
"THRA"
"NCOR2"
"PSMC5"
"ESR2"
"CCNE1"
"NR3C1"
"DD5"
"RB1"
"RB1"
"DD5"
"NR3C1"
"CCNE1"
"ESR2"
"PSMC5"
"NCOR2"
"THRA"
"NCOA1"
"NR2F1"
"PPARBP"
"NRIP1"
"HNF4A"
"PGR"
"BRCA1"
"VDR"
"RXRA"
"PPARGC1A"
"RAN"
"NCOA2"
"RARA"
"RAF1"
"RORA"
"NCOA3"
"PPARG"
"FHL2"
"AR"
"NCOA6"
"DAXX"
"THRAP4"
