# Supplementary material for: Network-Based Analysis of Affected Biological Processes in Type 2 Diabetes Models
Source: PLoS Genet. 2007 Jun 15;3(6):e96. doi: 10.1371/journal.pgen.0030096 (PMC1904360; doi:10.1371/journal.pgen.0030096)
Supplement: Supplementary file 5 [file pgen.0030096.sd006.doc]

We compiled Affymetrix CEL files and the sample annotations from the DGAP website

[1] and reprocessed each group of CEL files of the same array type. To calculate the expression values, we employed the latest algorithm from Affymetrix, named Probe Log Intensity ERror (PLIER) algorithm, which uses probe-specific characteristics across arrays to improve estimation. Multiple probe sets for the same transcript were averaged after excluding non-specific probe sets that ended with “_x_at”. For each experiment, we performed post hoc comparisons to the control group, as defined in the description of the experiment. Post hoc p-values were corrected for multiple comparisons within each experiment using Bonferroni's method. To integrate the data from the different species, we used Homologene Identifiers (version v39.2). This step reduces the number of available genes to 6652 for all the 19 experiments we collected. Of these 19 experiments, 16 were used in the analysis described in the paper. Refer to Dataset S1 for details on each experiment.

**References**

1. All raw data are available on the Diabetes Genome Anatomy Project website, [http://www.diabetesgenome.org](http://www.diabetesgenome.org/).
