# Supplementary material for: Network-Based Analysis of Affected Biological Processes in Type 2 Diabetes Models
Source: PLoS Genet. 2007 Jun 15;3(6):e96. doi: 10.1371/journal.pgen.0030096 (PMC1904360; doi:10.1371/journal.pgen.0030096)
Supplement: Supplementary file 6 [file pgen.0030096.sd007.doc]

**Assembling the nuclear receptor gene sets**

Nuclear receptors are well-established mediators in metabolic processes. One aim of the study was to determine whether subsets of nuclear receptors might be transcriptionally altered in insulin resistant or diabetic phenotypes. Three nuclear receptor genesets were assembled based on the nuclear receptor superfamily [1], labeled nuclear receptors from the HUGO Gene Nomenclature Committee [2] and the union of the two sets, from genes in the DGAP expression data and protein-protein interaction network. The sizes of the three genesets were 35, 32, and 49, respectively.

**Assembling the IS-HD gene set**

Because type 2 diabetes is characterized by insulin resistance, the insulin signaling pathway is a natural candidate for testing for alterations and associations with the disease phenotype. The 09-13-2005 version of the Human Protein Reference Database [3] annotated a set of 152 genes involved in insulin signaling. 91 of these genes were present in the DGAP expression data and protein-protein interaction network. This subset was labeled as the IS-HD geneset.

**Assembling the GO category gene sets**

GO categories associated with every gene in the DGAP expression data and protein-protein interaction network were determined from all levels of the GO hierarchy [4]. 2,353 unique GO categories, associated with at least one gene, were identified in this manner. For each of these categories, a geneset was assembled from corresponding, annotated genes. The genesets were then filtered by size, with the minimum and maximum thresholds (28 and 91, respectively) corresponding to the sizes of the smallest and largest of the non-GO category gene sets tested in this study, namely, the conservative retinol gene set (see **Assembling the retinol gene sets**) and IS-HD (see **Assembling the IS-HD gene set**), respectively. In this manner, 346 GO categories were retained after filtering.

**Assembling the retinol gene sets**

Previous studies have suggested a possible connection between retinol signaling and type 2 diabetes and related metabolic disorders [5-7]. An aim of the study was to determine whether sets of retinol signaling genes could be associated with insulin resistant or diabetic phenotypes.

Two genesets related to retinol metabolism and signaling were assembled from the Entrez Gene databank [8]. First, the database was searched with the query “(retinol OR retinoi*) AND human[orgn] AND alive”. The query returned 248 genes that were curated and assigned a code based on their relevance to retinol metabolism and signaling. A conservative retinol set of 28 genes was compiled from genes assigned codes 1-3. A liberal retinol set of 84 genes was compiled from genes assigned codes 1-7. Both retinol genesets were subset to include only the genes that appear in the DGAP expression data and protein-protein interaction network. The conservative retinol geneset was further subset to include only genes forming a connected subnetwork in the interaction network.

The codes and associated rules were ordered from most to least conservative. If more than one rule applies, the gene was assigned the code from the most conservative rule.

**Code	Code meaning**

1 The protein binds a retinoid compound
2 The protein is an enzyme (or its subunit) catalyzing conversion of a retinoid compound
3 The protein is a nuclear receptor that binds a retinoic acid receptor
4 The protein interacts with a protein defined by 1, 2 or 3
5 The gene is a direct transcription target of a retinoic acid receptor
6 Is a TF that regulates expression of a gene defined by 1, 2 or 3
7 The gene’s expression is regulated by a retinoid compound
8 Not related (none of the above applies)
48 Same as 4 but judged not related

**Assembling the NR-HD geneset**

The NR-HD geneset was assembled according to the following protocol.

1. A collection of 5 genesets was assembled. One was the conservative retinol geneset (see **Assembling the retinol genesets**). Another was the 49-member nuclear receptor geneset (see **Assembling the nuclear receptor genesets**). The remaining three were GO categories with a high score in GNEA — GO:0030518 (steroid hormone receptor signaling pathway), GO:0051427 (hormone receptor binding), and GO:0035257 (nuclear hormone receptor binding).
2. Each gene in the DGAP expression data and protein-protein interaction network was ranked by the number of HSNs to which they belong.
3. The top 68 genes appeared in at least one HSN.
4. Genesets were assembled from the top genes based on their number of appearances in the HSNs. For each number of appearances, a geneset was assembled from the top genes that appeared in at least that many HSNs.
5. The genesets were evaluated by GNEA. The geneset assembled from the top 30 genes was identified as the largest one with a significant p-value. This set was labeled as NR-HD.

**References**

[1] Robinson-Rechavi M, Garcia HE, Laudet V (2003) The nuclear receptor superfamily. J Cell Science 116:585-586.

[2] HUGO Gene Nomenclature Committee. <http://www.gene.ucl.ac.uk/nomenclature/>

[3] Human Protein Reference Database. [http://www.hprd.org](http://www.hprd.org/)

[4] Gene Ontology Consortium. [http://www.geneontology.org](http://www.geneontology.org/)

[5] Graham T, Yang Q, Bluher M, Hammarstedt A, Ciaraldi T, et al (2006) Retinol-Binding Protein 4 and insulin resistance in lean, obese, and diabetic subjects. N Engl J Med 354(24):2552-2563.

[6] Shulman A, Mangelsdorf D (2005) Retinoid X receptor heterodimers in the metabolic syndrome. N Engl J Med 353(6):604-615.

[7] Pfahl M, Chytil F (1996) Regulation of metabolism by retinoic acid and its nuclear receptors. Annu Rev Nutr 16:257-83.

[8] Entrez Gene Databank. http://www.ncbi.nlm.nih.gov/entrez/query.fcgi?db=gene
