# Supplementary material for: Network-Based Analysis of Affected Biological Processes in Type 2 Diabetes Models
Source: PLoS Genet. 2007 Jun 15;3(6):e96. doi: 10.1371/journal.pgen.0030096 (PMC1904360; doi:10.1371/journal.pgen.0030096)
Supplement: Supplementary file 7 [file pgen.0030096.sd008.doc]

**Assembling the general protein-protein interaction network**

The protein-protein interaction network was based on the proteins and physical interactions contained in the 09-13-2005 version of the Human Protein Reference Database (HPRD, [http://www.hprd.org](http://www.hprd.org/)). Only proteins in HPRD with valid accession ids were used. In total, 6,018 proteins and 19,890 physical protein-protein interactions were taken from HPRD to create the initial network. Network nodes were labeled by gene symbols.

**Determining condition-specific protein-protein interaction networks**

Condition-specific, protein-protein interaction networks were generated by mapping the relative mRNA expressions for each condition to the general protein-protein interaction network. The expression level for each gene was associated to the corresponding protein in the interaction network. Genes with no corresponding proteins in the general network were excluded. Similarly, proteins in the general network without a corresponding gene in the expression data were removed along with all associated interactions when determining the condition-specific networks.

The condition-specific, protein-protein interaction networks for the DGAP conditions each contained 3,643 nodes and 11,713 protein-protein interactions. Because 6652 total genes were present in the DGAP dataset, the percentage of DGAP genes represented by the condition-specific, protein-protein interaction networks was 54.77%, or roughly half.
