# Supplementary material for: Network-Based Analysis of Affected Biological Processes in Type 2 Diabetes Models
Source: PLoS Genet. 2007 Jun 15;3(6):e96. doi: 10.1371/journal.pgen.0030096 (PMC1904360; doi:10.1371/journal.pgen.0030096)
Supplement: Supplementary file 8 [file pgen.0030096.sd009.doc]

The Cytoscape plug-in jActiveModules was used to find a high-scoring network (HSN) for each condition, where the network score was a weighted average of the z-scores of the individual network members. The parameter values used to invoke the algorithm were *Start Temp* = 2.0, *End Temp* = 0.01, *Iterations* = 10^6^, and *Hubs* = 10. The *Hub-finding* and *Simulated Annealing* options were enabled while *Regional Scoring* was disabled. The algorithm was run twice in succession: The first time to find a high-scoring component in the mapped network, and the second time to find a high-scoring subcomponent within the high-scoring component. The output from the second run was taken as HSN. Such a recursive application of the algorithm was consistent with the discussion in the original paper [1] and the reader should refer to it for the full algorithm details as well.

**References**

[1] Ideker T, Ozier O, Schwikowski B, Siegel AF (2002) Discovering regulatory and signaling circuits in molecular interaction networks. Bioinformatics 18 Suppl 1:S233-40.
