# Supplementary material for: Network-Based Analysis of Affected Biological Processes in Type 2 Diabetes Models
Source: PLoS Genet. 2007 Jun 15;3(6):e96. doi: 10.1371/journal.pgen.0030096 (PMC1904360; doi:10.1371/journal.pgen.0030096)
Supplement: Supplementary file 9 [file pgen.0030096.sd010.doc]

A geneset score was assigned a p-value by comparing it against a random score distribution.

For GNEA, 10,000 genesets of the same size as the original were generated by a random walk through the condition-specific, protein-protein interaction network as follows:

1. Initialize the geneset with a randomly selected node in the interaction network, with equal probability of selection for all nodes.
2. Determine the neighbors of the current subgraph in the interaction network.
3. Randomly select one of the neighbors and add it to the geneset.
4. Repeat (2) – (4) until the geneset reaches the proper size.

Each random geneset was scored by GNEA and a distribution of all 10,000 scores was subsequently generated.

The protocol for DEA was simpler: For a given size *k*, randomly select *k* genes from the DGAP expression data to form each of the 10,000 random genesets. Each geneset was scored by DEA to generate the random score distribution.
